# Seed Embryo Development Is Regulated via an *AN3-MINI3* Gene Cascade

**DOI:** 10.3389/fpls.2016.01645

**Published:** 2016-11-03

**Authors:** Lai-Sheng Meng, Yi-Bo Wang, Gary J. Loake, Ji-Hong Jiang

**Affiliations:** ^1^The Key Laboratory of Biotechnology for Medicinal Plant of Jiangsu Province, School of Life Science, Jiangsu Normal UniversityXuzhou, China; ^2^Centre for Transformational Biotechnology of Medicinal and Food Plants, Jiangsu Normal University – The University of EdinburghXuzhou, China; ^3^School of Bioengineering and Biotechnology, Tianshui Normal UniversityTianshui, China; ^4^Institute of Molecular Plant Sciences, School of Biological Sciences, The University of EdinburghEdinburgh, UK

**Keywords:** *ANGUSTIFOLIA3* (*AN3*), MINISEED*3* (*MINI3*), seed mass, embryo cell elongation, embryo cell division, *Arabidopsis*

## Abstract

In agriculture, seed mass is one of the most important components related to seed yield. *MINISEED3* (*MINI3*) which encodes the transcriptional activator WRKY10, is thought to be a pivotal regulator of seed mass. In *Arabidopsis* SHORT HYPOCOTYL UNDER BLUE1 (SHB1) associates with the promoter of *MINI3*, regulating embryo cell proliferation (both cell division and elongation), which, in turn, modulates seed mass. Furthermore, the recruitment of SHB1 via MINI3 to both its cognate promoter and that of *IKU2* implies a two-step amplification for countering the low expression level of *IKU2*, which is thought to function as a molecular switch for seed cavity enlargement. However, it is largely unknown how embryo cell proliferation, which encompasses both cell division and elongation, is regulated by SHB1 and MINI3 function. Here, we show that a loss of function mutation within the transcriptional coactivator ANGUSTIFOLIA3 (AN3), increases seed mass. Further, AN3 associates with the *MINI3* promoter *in vivo*. Genetic evidence indicates that the absence of MINI3 function suppresses the decrease of cell number observed in *an3-4* mutants by regulating cell division and in turn inhibits increased cell size of the *an3-4* line by controlling cell elongation. Thus, seed embryo development is modulated via an *AN3-MINI3* gene cascade. This regulatory model provides a deeper understanding of seed mass regulation, which may in turn lead to increased crop yields.

## Introduction

*ANGUSTIFOLIA3* (*AN3*)/*GRF-INTERACTING FACTOR1*, encodes a homolog of the human transcription coactivator, synovial sarcoma translocation protein (SYT) (Horiguchi et al., 2005). *AN3*, a member of a small gene family in *Arabidopsis* (Horiguchi et al., 2005), has been implicated in modulating cell proliferation, adaxial/abaxial determination in leaf primordia and establishing cotyledon identity (Horiguchi et al., 2005, 2011; Kanei et al., 2012). AN3/GIF1 has been shown to function as a component of a complex implicated in modulating the growth and shape of leaf blades and petals (Kim and Kende, 2004). *AN3* expression is restricted to within mesophyll cells and is not detected within epidermal cells (Horiguchi et al., 2011).

Seed mass is modulated through three major components – embryo, endosperm and seed coat – derived from different cells of the ovule and with distinct complements of maternal and paternal genomes. In angiosperms, seed development involves a double-fertilization process in which two polar cells fuse to form the central cell before fertilization. Thus, one sperm cell fuses with the egg cell and the other fuses with the diploid central cell to form the triploid endosperm (Lopes and Larkins, 1993). When seed maturity is completed, the seed possesses only a single layer of endosperm cells and the maternal integument forms the seed coat. The embryo is surrounded by the endosperm, which in turn is surrounded by the maternal seed coat. Therefore, the coordinated growth of maternal sporophytic and zygotic tissues determines seed mass.

The accumulating evidence suggests that seed mass is genetically regulated. The *apetala2* (*ap2*) mutant has enhanced seed mass due to increases of both embryonic cell size and cell number. DA1 which functions as a growth-repressor, is a ubiquitin receptor with two ubiquitin interaction motifs. Further, the *da1-1* mutant exhibits large seed mass due to alterations in the maternal integuments of ovules (Li et al., 2008). Mutants in an enhancer of *da1-1, EOD1*, encoding the E3 ubiquitin ligase, BIGBROTHER (BB) (Disch et al., 2006; Li et al., 2008), synergistically increase the seed mass phenotype of *da1-1*. Thus, revealing that *DA1* acts synergistically with *EOD1/BB* to regulate seed size.

A triple mutant of the three cytokinin receptors produces twice the seed mass as the corresponding wild-type line. In this context, it has been proposed that cytokinin might regulate embryo mass through a maternal and/or endosperm based mechanism (Hutchison et al., 2006; Riefler et al., 2006). *Arabidopsis auxin response factor2* (*arf2*) mutant plants have extra cell proliferation in the integuments of the mutant ovules and therefore have enlarged seed coats and ultimately enhanced seed mass (Schruff et al., 2006). *MINISEED3* (*MINI3*) which encodes a WRKY transcription factor, is expressed in both endosperm and embryo (Luo et al., 2005), while *HAIKU2* (*IKU2*), a leucine-rich repeat receptor kinase gene, is predominantly expressed in the endosperm but its expression is absent from the embryo (Luo et al., 2005). Both *iku2* and *mini3* plants exhibit decreased seed mass and their seed phenotypes are determined through the genotype of either embryo or endosperm (Luo et al., 2005). Therefore, IKU1, IKU2, and MINI3 function in the same pathway of seed development (Garcia et al., 2003; Luo et al., 2005; Wang et al., 2010).

The gain-of-function mutant, SHORT HYPOCOTYL UNDER BLUE 1 (SHB1), exhibits a large seed mass as a result of increased cell number and enhanced cell size (Zhou et al., 2009). In contrast, the *mini3* mutant has a small seed mass as a result of a decreased cell number (Luo et al., 2005; Zhou et al., 2009). Furthermore, the binding of SHB1 to both the *MINI3* and *IKU2* promoters suggests a two-step amplification for countering the low expression level of *IKU2*, which is thought to function as a molecular switch for seed cavity enlargement (Zhou et al., 2009; Kang et al., 2013). Recently, it has been proposed that ABA negatively regulates SHB1 transcription through the action of the basic leucine zipper protein, ABI5, which directly binds to ABRE *cis*-elements within the SHB1 promoter, which are required for the regulation of seed development (Cheng et al., 2014). Thus, ABI5-SHB1-MINI3-IKU1 forms a gene cascade that negatively controls embryo cell proliferation and by extension also seed mass. However, it is largely unknown how embryo cell division and elongation is fine-tuned and consequently, how in detail seed mass is regulated. Uncovering this mechanism will provide a better understanding of seed mass regulation.

In the present study, we have found that the loss-of-function mutant, *an3-4*, exhibited increased seed mass. Further, molecular analysis indicated that *MINI3* was a target gene of AN3. Genetically, AN3 acted upstream of MINI3: mutation of *MINI3* inhibited the decrease of cell number (cell division) in the *an3-4* mutant and suppressed the increase of cell size (cell elongation) during embryo development of *an3-4* plants. Thus, an *AN3-MINI3* gene cascade may regulate seed embryo development by amplification (cell division and cell elongation). Our proposed model provides a greater understanding of seed mass regulation, which may in turn help guide approaches to increase crop yields.

## Materials and Methods

### Plant Materials and Growth Conditions

The *an3-4* and *mini3-2* mutants were described previously (Horiguchi et al., 2005; Zhou et al., 2009). *shb1* (SALK_128406), *grf1* (SALK_001350), *mini3-2*(SALK_050364), *iku2-4* (SALK_073260), and *ap2* (SALK_071140) were in the Col background and obtained from the ABRC (Ohio State University, Columbus). The *an3 mini3* double-mutant was obtained from F_2_ seedlings of *an3-4* ×*mini3-2* that had a narrow rosette leaf phenotype of plants grown in white light (the *an3-4* phenotype for homozygous lines; Horiguchi et al., 2005). The *an3 mini3* mutant was confirmed in *F*_3_ by PCR genotyping of *MINI3* using the gene-specific primers for *MINI3* described by Zhou et al. (2009). *grf1* (SALK_001350) homozygous lines were obtained through herbicide selection for three or more generations and analysis of segregation ratios. Absence of gene expression in the mutant was verified by RT-PCR. *pMD111-Pro MINI3:GFP* (*MINI3 pro:GFP*) were introduced into the *35S:AN3* background by *Agrobacterium* mediated transformation of *35S:AN3* homozygous plants. Transformants were selected on hygromycin B for three or more generations and segregation ratios analyzed. This expression levels of transgenes in these transgenic lines was confirmed. The resulting seeds were subjected to 4°C for 3 days, and then sown onto solid Murashige and Skoog (MS) medium supplemented with 1% sucrose at pH 5.8 and 0.8% agar. The seedlings grown on agar were maintained in a growth room under 16/8 h light/dark cycles with cool white fluorescent light at 21 ± 2°C. Plants grown in soil-less media were maintained in a controlled environment growth room under 16/8 h light/dark cycles with cool white fluorescent light at 21 ± 2°C under constant light (120 μmol⋅m^-2^⋅s^-1^ light from a mixture of fluorescent and incandescent bulbs).

### Semi-Quantitative RT-PCR (SQ-RT-PCR) and Quantitative PCR

Total RNA was extracted from tissues indicated in the figures by the TRIZOL reagent (Invitrogen), as described by Meng et al. (2015a,b). SYBR [Sangon Biotech (Shanghai) Co., Ltd] was used to monitor the kinetics of PCR product in real-time RT-PCR (Meng et al., 2015a,b). Briefly, first-strand cDNA samples were produced from total RNA samples via reverse transcription using an AMV reverse transcriptase first-strand cDNA synthesis kit (Life Sciences, Promega) and were used as templates for RT-PCR–based gene expression analysis. For qRT-PCR analysis, after RNA isolation, reverse transcription was performed according to the manufacturer’s protocol (M-MLV reverse transcription system; Promega), followed by qPCR analysis to determine the gene expression level (Bio-Rad iQ5). The oligonucleotide primer sequences used to amplify specific cDNAs are described in **Supplementary Table S2**.

For analyzing *AP2, SHB1, IKU2*, and *MINI3* expressions in 10–12 days-after-pollination (DAP) siliques of *an3-4, grf1* and wild-type (whole siliques), the primers were employed as shown in **Supplementary Table S2**. For analyzing *AN3* expression in *mini3-2, shb1* (SALK_128406), *iku2-4* (SALK_073260), and *ap2* (SALK_071140) 10–12 DAP siliques (whole siliques), the primers were employed as shown in **Supplementary Table S2**. For SQ-RT-PCR analysis, the primers were utilized as shown in **Supplementary Table S2**. These experiments were all repeated at least twice with similar results.

### Plasmid Constructs

By promoter analysis, an *AN3* (At5g28640) promoter–*GUS* construct was produced through inserting a ∼2.0-kb promoter fragment (*pGWC* vector, Gateway, Lei et al., 2007), amplified by specific primers (**Supplementary Table S2**). These constructs were transferred into *pCB308R*, as described by Lei et al. (2007). For obtaining *pCB2004-35S-AN3* in the associated plasmid, primers were outlined in **Supplementary Table S2**. For obtaining *pMD111-MINI3:GFP* in the associated plasmid, the relevant primers are listed in **Supplementary Table S2**.

### ChIP Assay

Transgenic lines over-expressing *35S:AN3:3XGFP* (Kawade et al., 2010) in an *an3-4* genetic background and transgenic *35S:GFP* lines were used in this assay. ChIP was performed using transgenic plants 10–12 DAP whole siliques as materials. The details of the ChIP process were described by Saleh et al. (2008). Briefly, *Agrobacterium* with a GFP construct was introduced into *an3-4* 10–12 DAP siliques using a leaf disk method (Meng et al., 2016). Two days after transformation, these transgenic materials (*an3-4-GFP*) were used for ChIP. 1.5 g of 10–12 DAP siliques of *35S:AN3-3XGFP* plants were harvested and then fixed in 1% formaldehyde for 10–15 min in vacuum and neutralized with 0.125 M Gly in vacuum for an additional 5 min. After washing twice with cold, sterile water, these tissues were ground in liquid nitrogen. Nuclei were isolated and sonicated. Sonicated chromatin supernatant (250 μL) was diluted to 3, and 20 mL of protein *A*-agarose bead (Upstate) was added for preclearing at 4°C for 1–2 h. Then, the chromatin was divided into two 1.5-mL aliquots. Five microliters of mouse GFP and HA tag-specific monoclonal antibody (Sigma–Aldrich) was added to one tube. After incubating at 4°C overnight, beads were washed with low-salt wash buffer, high-salt wash buffer and TE (for Tris– EDTA buffer [10 mM Tris, 1 mM EDTA, pH8.0]) buffer. Elution and reverse cross-linking were performed as previously described (Saleh et al., 2008). Eluates were treated with Proteinase K (10 mg/mL; Sigma–Aldrich) and RNase for 3.0 h at 45°C, then phenol/chloroform extracted and ethanol precipitated with the aid of 20 μg of glycogen. The purified DNA was resuspended in 50 mL of water. The enrichment of DNA fragments was measured by qPCR using primers listed in **Supplementary Table S2** online.

### GUS Assay

Plants were incubated at 37°C for 8–10 h in a buffer mix of 0.4 mM of K3Fe(CN)6/K4Fe(CN)6, 1 mM X-gluc, 60 mM NaPO4 buffer and 0.1% (v/v) Triton X-100. Chlorophyll was removed using sequential washes for 30 min with 30, 50, 70, 90, and 100% ethanol, as described by Meng (2015) and Meng and Yao (2015).

### Protein Analysis

Total protein extracts from 15 mature seeds of wild-type, *an3-4* mutants and transgenic plants harboring the *35S:AN3:3XGFP* transgene in an *an3-4* background were fractionated on 8% SDS-PAGE and stained with Coomassie Brilliant Blue as described by Ohto et al. (2005).

### Confocal Laser Scanning for GFP Imaging

Subcellular localization of MINI3: Green Fluorescent Protein (GFP) fusion proteins was undertaken in the abaxial epidermis of 8-day-old transgenic plants harboring *pMD111-MINI3:GFP*. An Olympus IX-70 microscope^1^ was used for detecting GFP expression using λ = 488 nm and λem = 510 nm. The sections were photographed under a confocal laser scanning microscope.

### Cytological Experiments

Average seed weight was tested through weighing mature dry seeds in batches of 100 using an electronic analytical balance (Mettler, Toledo). Measurements of cotyledon area were made through scanning these organs to form a digital image and then calculating area using Image J software. Mature seeds were photographed under relevant magnification using a HIROX three-dimensional video microscope.

Mature dried seeds were imbibed for 60–100 min and dissected under microscope for isolating mature embryos. The embryos were incubated overnight in buffer (30 mM sodium phosphate, pH 7.0, 10 mM EDTA, 1% Triton X-100 and 1% DMSO) at 37°C, fixed for 1 h in buffer (FAA with 10% formalin, 5% acetic acid and 45% ethanol) and 0.01% Triton X-100, and dehydrated with an ethanol series, as described by Ohto et al. (2005). The embryos were then treated for 1–2 h in Hoyer’s buffer (3:0.8:0.4 of chloral hydrate: water: glycerol). A HIROX three-dimensional video microscope, under relevant magnification, was used to observe the treated embryos. The cell size of cotyledon embryos and the cell size of globular stage embryos were measured using Image J software.

## Results

### *an3-4* Plants Have Increased Seed Mass

While investigating drought tolerance exhibited by *an3-4* plants, we observed this line had larger cotyledons than wild-type (Col-0). To confirm whether these large cotyledons were due to a lack of AN3 protein activity, we investigated the phenotype of *an3-4* complemented lines (Kanei et al., 2012). These lines harboring a *35S:AN3* transgene in an *an3-4* genetic background exhibited similar cotyledon size to Col-0 (**Supplementary Figures S1A,B**). Significantly, AN3 interacts with GRF1 to regulate leaf development (Horiguchi et al., 2005). However, a *GRF1* loss-of-function mutant showed similar cotyledon size relative to wild-type (**Supplementary Figures S1A,B**), implying GRF1 may not be involved in the regulation of seed mass. Moreover, while Ferjani et al. (2007) reported that the *an3-4* mutant presented both larger cotyledons and embryos than wild-type (Col-0), an impact on seed mass was not highlighted. Taken together our data suggests that the *an3-4* plants have larger cotyledons relative to wild-type (Col-0).

**FIGURE 1:**
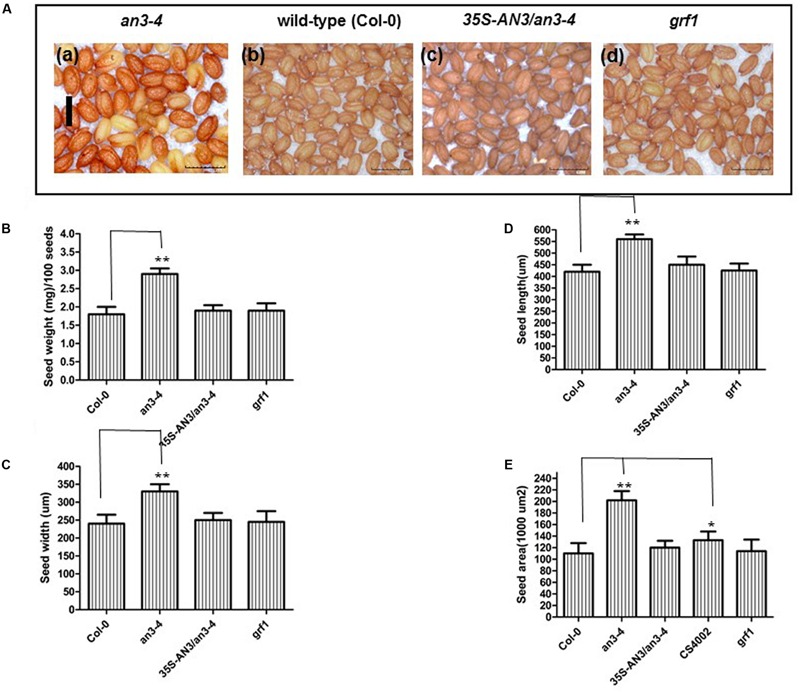
***an3-4* mutant plants have increased seed mass. (A)**. Representative mature dry seeds of *an3-4*, Col-0, *35S-AN3*/*an3-4*, and *grf1* plants grown on solid MS medium with 1% sucrose, respectively. Bar = 0.1 mm for (a)–(d). **(B–E)** Bar graph showing the difference in average seed weight/100 seeds **(B)**, seed width **(C)**, seed length **(D)**, and seed area **(E)** between *an3-4*, Col-0, *an3-4*+*35S-AN3* and *grf1* seeds. Data are means ± SD from at least 10 independently propagated lines of *an3-4*, WT, *an3-4*+*35S-AN3* and *grf1* (^∗^*P* < 0.05; ^∗∗^*P* < 0.01; in B, *n* = 4; in **C–E**, *n* = 30).

Further the seeds of self-pollinated homozygous *an3-4* mutant plants weighed ∼60% more than Col-0 and *an3-4* complemented lines. Also, they exhibited a pale orange coloration relative to wild-type [**Figures 1A(a–c),B**]. The area, length and width of these seeds were all enhanced in *an3-4* plants relative to wild-type [**Figures 1A(a–c),C–E**]. However, a *GRF1* loss-of-function mutant was similar to wild-type for these traits (**Figures 1A–E**). Interestingly, aborted seeds can be observed in the *35S:AN3* complemented line, which may be a result of the imperfect activity of the *35S* promoter in embryonic tissues [**Figure 1A(c)**]. AN3 is also a positive regulator of anthocyanin biosynthesis (Meng, 2015; Meng and Liu, 2015) and the *35S:AN3 an3-4* line largely restored anthocyanin accumulation in *an3-4* plants [**Figure 1A(c)**]. Thus, AN3 is a regulator of both seed mass and anthocyanin biosynthesis, in a similar fashion to *transparent testa glabra2* (*ttg2*) (Garcia et al., 2005). Collectively these data suggest that *AN3* negatively regulates seed mass.

### *an3-4* Mutant Plants Have Lower Seed Yield

**FIGURE 2:**
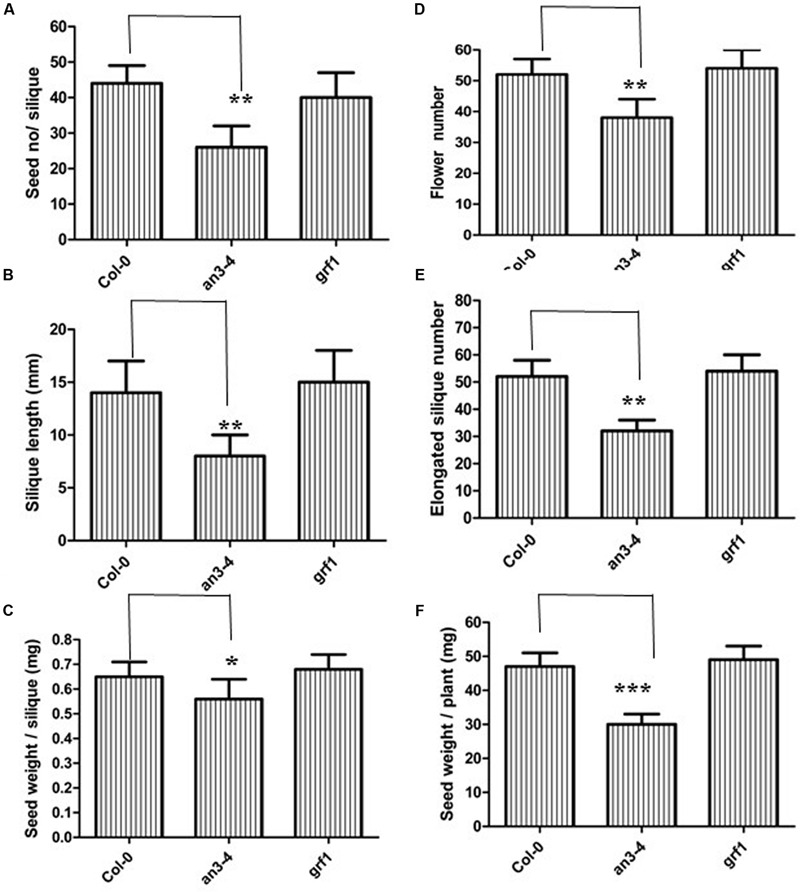
***an3-4* mutant plants have low seed yield.** For the given plant genotypes representative seed number/silique **(A)**, silique length **(B)**, seed weight/silique **(C)**, flower number **(D)**, elongation silique number **(E)**, and seed weight/plant **(F)** are shown, respectively. Data are means ± SD from at least 10 independently propagated Col-0, mutant lines or independent transgenic lines (^∗∗∗^*P* < 0.001, ^∗∗^*P* < 0.01, ^∗^*P* < 0.05, respectively; in **A**, *n* = 12; in **B**, *n* = 10; in **C**, *n* = 15; in **D**, *n* = 6; in **E**, *n* = 6; in **F**, *n* = 6).

Understanding the molecular machinery underpinning the control of seed mass may help guide future improvements in crop yields. A large seed mass is associated with an altered seed yield, for example, *ap2* plants exhibited increased seed mass but gave a lower seed yield (Ohto et al., 2005). However, *da1* plants had a larger seed mass and conversely, a higher seed yield (Li et al., 2008). Thus, we determined the relationship between increased seed mass in *an3-4* plants and seed yield. We therefore analyzed the seed/silique number, silique length, seed/silique weight, flower number, elongated silique number, and total seed weight. While the *an3-4* mutant exhibited increased seed mass, those values associated with total seed yield all declined (**Figure 2F**). Thus, seed/silique number, silique length, and seed/silique weight were all reduced in the *an3-4* mutant line relative to Col-0 plants (**Figures 2A–C**). However, these parameters were not significantly different in *grf1* plants relative to wild-type (**Figures 2A–F**). Due to a deficiency in reproductive development, the decrease in elongated silique number in *an3-4* mutant plants relative to flower number resulted in a reduced seed yield (**Figures 2D,E**). Therefore, we determined flower morphology and found that the petals and leaf blades of *an3-4* plants were smaller than those of wild-type (**Supplementary Figure S2**; Horiguchi et al., 2005). Occasionally, flowers on the primary inflorescences of *an3-4* plants did not open in a similar fashion to wild-type. Further, stamens of *an3-4* plants were dramatically shorter compared with wild-type (the frequency: 28.0 ± 2.2; **Supplementary Figure S2**). Also, in the *an3-4* line, the dehiscence of anthers was severely delayed (**Supplementary Figure S2**). These observations might partially explain why the elongated silique number in *an3-4* mutant plants was decreased relative to flower number. In aggregate, these data imply the, lack of AN3 activity led to increased seed mass but resulted in a lower seed yield.

### The Increased Seed Mass of *an3-4* Plants May Be Related with Low Fertility and the Absence of AN3

An allocation of extra resources for fewer seeds produced, due to decreased fertility, can also result in larger seed mass (Ohto et al., 2005). Thus, we determined whether increased seed mass in *an3-4* plants was caused by an allocation of extra resources. We hand-pollinated five flowers from the primary inflorescences of wild-type, *an3-4* plants and a male-sterile mutant (*CS4002*). Manual pollination ensured that all siliques had similar numbers of seeds. Thus, flowers were pollinated using pollen from the same genotype, but wild-type pollen was used as the donor in male-sterile plants. After maturity, each male-sterile mutant produced five siliques. On average, the seed weight was ∼18% higher when derived from the male-sterile plant compared to wild-type (**Figure 1E**), suggesting that reduced fertility can enhance seed weight. However, the average seed weight of the *an3-4* mutant was ∼60% higher than that of the wild-type (**Figure 1E**), indicating that the increased seed mass exhibited by *an3-4* plants may be partially associated with low fertility and in addition to an absence of AN3 function.

### The Large Seeds of *an3-4* Plants Are Due to Increased Embryo Cell Size

**FIGURE 3:**
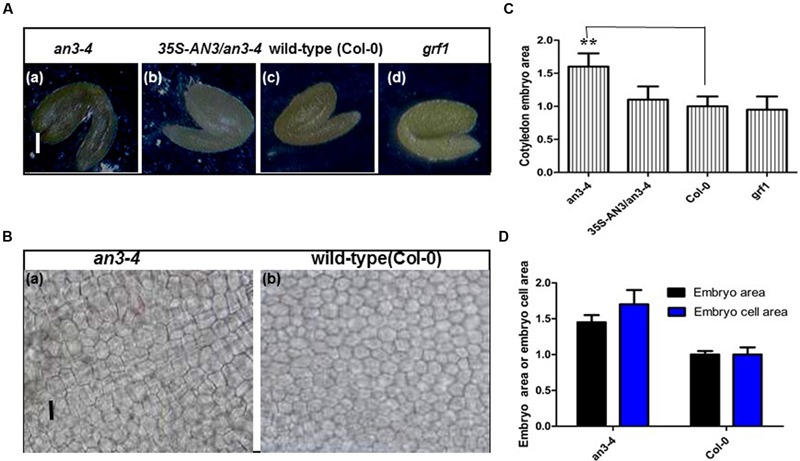
***an3-4* mutant plants have large embryos caused by increased embryo cell size. (A)** Representative mature embryos of *an3-4, 35S-AN3*/*an3-4*, Col-0, and *grf1* plants grown on solid MS medium with 1% sucrose, respectively. Bars = 100 μm. **(B)** Representative epidermal cell layer derived from the central region of cotyledon embryos from *an3-4* mutant and Col-0 seeds, respectively. Bars = 10 μm. **(C)** Bar graph showing the difference in cotyledon embryo area between *an3-4, 35S-AN3*/*an3-4*, Col-0, and *grf1* plants. Data are means ± SD from at least five independently propagated Col-0, mutant lines and independent transgenic lines (^∗∗^*P* < 0.01; *n* = 10). Col-0 is set as 1.0. **(D)** Bar graph showing the difference in embryo area and embryo cell area between *an3-4* and Col-0 plants. Col-0 is set as 1.0. Data are means ± SD from at least five independently propagated Col-0 and mutant lines (embryo area, *n* = 12; embryo cell area, *n* = 28).

The embryo constitutes the major volume of a mature seed in *Arabidopsis* and thus changes in embryo cell size impact seed mass. Thus, we isolated and visualized mature embryos originating from the self-pollinated homozygous seeds of wild-type, complemented *an3-4* lines and *an3-4* plants. The *an3-4* mature embryos were larger than those of wild-type and complemented *an3-4* lines (**Figures 3A,C**). However, these parameters were similar in *grf1* plants relative to wild-type (**Figures 3A,C**). Ferjani et al. (2007) showed that the *an3-4* mutant presented larger embryos than wild-type (Col-0), but this was not mentioned by these authors. Cytological observations indicated that the average area of cotyledon embryos from *an3-4* plants was ∼1.40 times that of wild-type (**Figures 3B,D**). On average, the epidermal cell size of *an3-4* embryos was ∼1.70 times that of wild-type (**Figures 3B,D**). These data (i.e., 1.40/1.70 = 0.8) indicated that the increased embryo size exhibited by *an3-4* plants was due to embryo cell elongation. AN3 positively regulates leaf cell proliferation (Horiguchi et al., 2005). The larger epidermal cells in *an3-4* embryos are probably a result of a phenomenon termed compensation, in which a decrease in cell number triggers an increase in mature cell size (Ferjani et al., 2007; Fujikura et al., 2007). This compensation-induced cell enlargement is mostly independent from endoreduplication, however, the underpinning mechanism is unknown (Ferjani et al., 2007; Fujikura et al., 2007). Furthermore, the increased cell size phenotype (termed compensation) is a secondary effect triggered by impaired cell proliferation (Fujikura et al., 2009). Therefore, the absence of AN3 resulted in larger embryo cells which resulted in both larger embryos and seeds.

### Seeds from *an3-4* Plants Exhibited an Increased Protein Content Relative to Wild-Type Seeds

**FIGURE 4:**
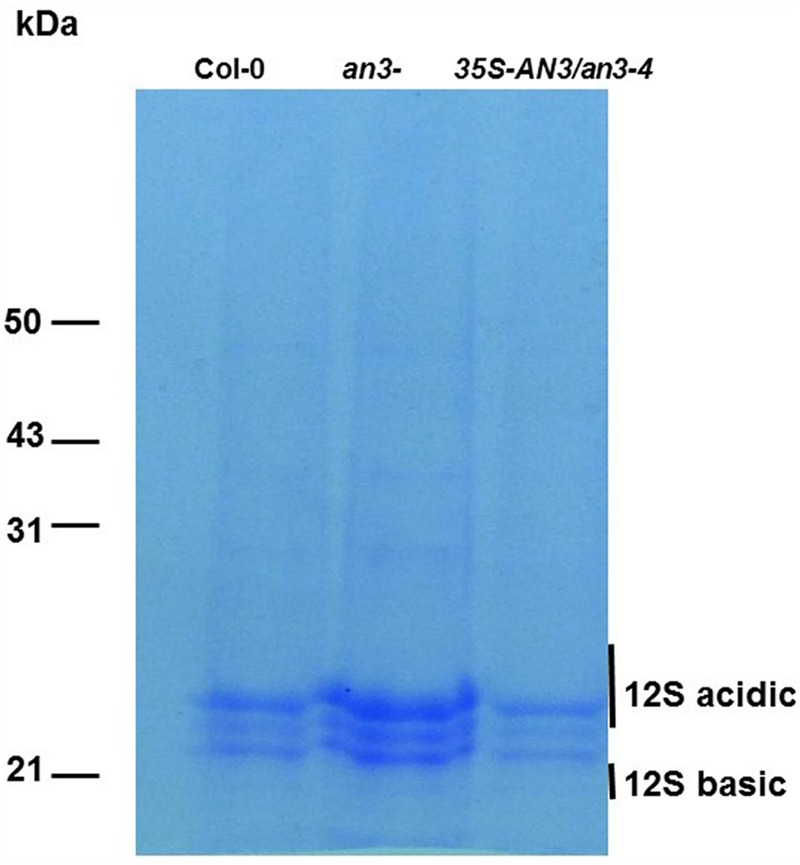
***an3-4* protein analysis.** *an3-4* seeds contain more protein than wild-type seeds. Protein extracts from 15 wild-type and *an3-4* seeds were fractionated on an 8% SDS/polyacrylamide gel and stained. Molecular mass markers are shown to the left of the gel.

Increased embryo cell size at the mature phase of seeds promotes accumulation of storage reserves (Ohto et al., 2005). Two major classes of *Arabidopsis* seed storage proteins are the 12S cruciferins and the 2S albumins (Heath et al., 1986; Pang et al., 1988). Therefore, we prepared protein extracts from 15 seeds each derived from an*3-4*, wild-type and complemented *an3-4* lines. The 12S and 2S protein content form these seeds was then determined by SDS-PAGE. 12S proteins were obviously in greater abundance in seeds from the *an3-4* line compared to wild-type, whereas levels of this protein did not significantly differ between complemented *an3-4* lines and wild-type (**Figure 4**). However, the 2S albumin proteins were not retained in the 8% SDS-PAGE gel (**Figure 4**). These results suggest that 12S protein accumulation is increased in the absence of AN3 activity. This implied that the overall proportion of individual proteins were not affected by lack of AN3 activity.

### AN3 Negatively Regulates *MINI3* Expression during Reproductive Growth

**FIGURE 5:**
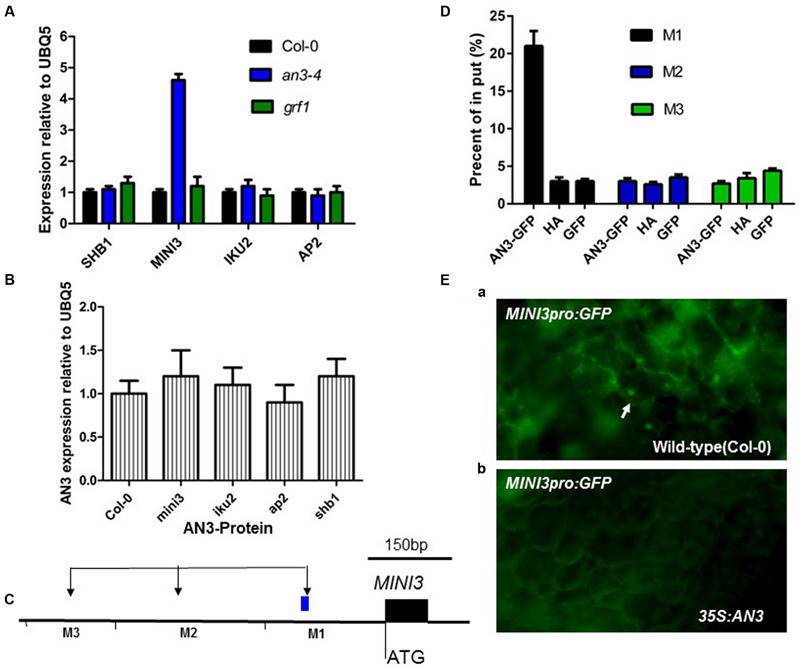
**AN3 Associates with the *MINI3* promoter *in vivo* during seed development. (A)** Bar graph exhibiting the expression difference in *SHB1, MINI3, IKU2*, and *AP2* between the WT (Col-0), *an3-4*, and *grf1* 10–12 DAP siliques. **(B)** Bar graph showing the difference in expression of AN3 between WT, *mini3-2, iku2-4* (SALK_073260), *ap2* (SALK_071140), and *shb1* (SALK_128406) 10–12 DAP siliques. Three biological replicates and two technical repeats were undertaken. Error bars represent SD. **(C)** Schematic diagram of *MINI3* and three amplicons initiating from the ATG of *MINI3*: M1, M2, and M3 used for ChIP analysis. **(D)** Bar graph showing AN3 association with the *MINI3* promoter. ChIP was performed to analyze the *in vivo* interaction of AN3 with the *MINI3* promoter. Input was chromatin before immunoprecipitation. Anti-GFP antibody was used for precipitating chromatin associated with 35S-AN3-3XGFP and 35S-GFP. Anti-HA antibody was used for precipitating chromatin associated with 35S-AN3-3XGFP. Anti-GFP antibody was used for precipitating chromatin associated with 35S-AN3-3XGFP. Anti-GFP antibody was used for precipitating chromatin associated with 35S-GFP. The *MINI3* promoter region associated with AN3 was amplified by qPCR using *MINI3* promoter-specific primers for distinct regions. Three biological replicates were performed. Error bars represent SD. **(E)** *MINI3pro: GFP* in WT (Col-0) (a) and 35S:AN3 (b) cotyledons. White arrows point to GFP-positive nuclei.

It is well established that *IKU2, MINI3, AP2*, and *SHB1* are involved in modulating seed mass (Luo et al., 2005; Ohto et al., 2005; Zhou et al., 2009). Therefore, we performed real-time PCR experiments to determine whether *AN3* modulated expression of these genes using siliques 10–12 DAP from *an3-4* and wild-type plants. *MINI3* transcript levels in *an3-4* plants were 4.6 times greater than those found in wild-type (*P* < 0.01, two-tailed Student’s *t*-test) (**Figure 5A**). Conversely, the transcript levels of complemented *an3-4* lines were 0.6 times that of wild-type plants (*P* < 0.05). However, the expressions of *IKU2, AP2* and *SHB1* were not significantly different between *an3-4* lines or Col-0 plants (**Figure 5A**). Also, the expressions of *IKU2, AP2, MINI3*, and *SHB1* were not significantly different between the *grf1* mutant and Col-0 plants (**Figure 5A**). Moreover, *AN3* expression did not significantly differ between Col-0 plants and *ap2, shb1, mini3*, and *iku2* mutants (**Figure 5B**). Thus, while *MINI3* expression was increased in an *an3-4* genetic background, *AN3* expression was unaltered in *mini3* plants relative to the wild-type line. Collectively, these data suggest that AN3 might negatively regulate *MINI3* at the transcriptional level.

### AN3 Represses *MINI3* Promoter Activity

To confirm that AN3 regulated *MINI3* expression, we performed chromatin immunoprecipitation (ChIP) analysis using 10–12 DAP siliques of transgenic plants harboring a *35S:AN3:3XGFP* transgene. The *35S:GFP* lines were used as a negative control. Chromatin associated with AN3–GFP and GFP was immunoprecipitated with an Anti-GFP antibody. Real-time PCR analysis was then performed with primers specific for different regions of the *MINI3* promoter (**Figure 5C**). Regions of M1 (-210 to -370 bp) primers produced a large amount of PCR product (21% of Input), whereas less PCR product (3 or 2.7% of Input in M2 or M3, respectively) was detected in the region of M2 (-822 to -1053 bp) and M3 (-1258 to -1463 bp) primers (**Figure 5D**). Anti-HA used as a negative control resulted in significantly less PCR product, 3, 2.6 or 3.4% of input in the M1, M2, or M3, respectively (**Figure 5D**). Using the *35S:GFP* lines as materials, less PCR product (3, 3.5, and 4.4% of Input in M1, M2, and M3, respectively) was detected in the region of M1, M2, and M3 primers (**Figure 5D**). At 8 days post-germination (dpg), wild-type plants expressed a transgene comprised of the *MIN3* promoter adjacent to a gene encoding the Green Florescent Protein (*MINI3pro:GFP*) (13 of 15 plants). However, the *MINI3pro:GFP* is not expressed in a *35S:AN3* background (0 of 15 plants) [**Figures 5E(a,b)**], indicating that AN3 is a major factor repressing the *MINI3* promoter. These results suggest that AN3 is associated with the *MINI3* promoter for the control of seed mass. Moreover, AN3 was unable to bind directly to the *MINI3* promoter sequences in a gel mobility shift assay (our unpublished data), which is consistent with the notion that AN3 is a transcriptional coactivator.

### *AN3* Expression Pattern

**FIGURE 6:**
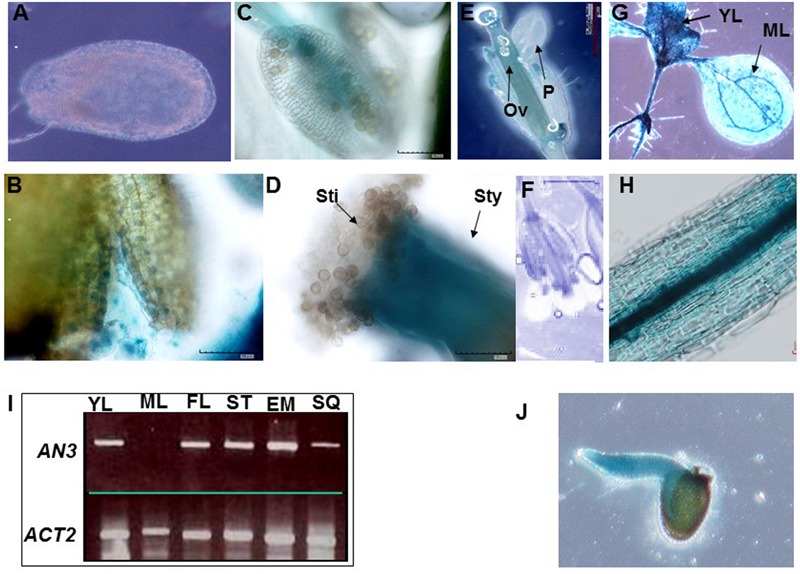
***AN3* expression analysis.** Expression of *AN3pro:GUS* in the seed coat **(A)**, testa **(B)**, mature pollen and stamens **(C)**, stigma and style **(D)**, flowers **(E)**, and silique **(F)**, seedlings with young leaves and mature leaves **(G)**, stems **(H)**, and embryos **(J)**. Ov, ovule; P, petal; Sti, stigma; Sty, style. **(I)** *AN3* expression analysis in several organs by SQ-RT-PCR. YL, young leaves; ML, mature leaves; FL, flower; ST, stem; EM, embryo; SQ, silique.

To further explore AN3 function, we examined the expression pattern of the β-glucuronidase (GUS) reporter gene driven by a native *AN3* promoter. Analysis of *Arabidopsis* transformed with an approximately 2.0 kb *AN3* promoter–GUS reporter gene fusion (*AN3pro:GUS*) showed that *AN3pro:GUS* was expressed in the seed coat, testa, stamens, petals, siliques, young leaves, stems and embryos, but not in endosperms, mature leaves and mature pollen grains (**Figures 6A–J**). Consistent with this, RT-PCR results indicated the presence of *AN3* transcripts in the corresponding organs (**Figure 6I**). These results implied that *AN3* is expressed in root, shoot, and flower organs; indicating *AN3* is widely expressed. Moreover, Kanei et al. (2012) observed that *AN3* can be strongly expressed in the globular, heart, torpedo, and cotyledon embryo. Thus, *AN3* might function during the whole of embryo development. And Lee et al. (2014) suggested that *AN3* might be expressed in the endosperm.

When an organ undergoes growth by proliferation, a fundamental event is the duration of cell proliferation, which generates a sufficient number of cells to support further growth and finally determines the overall size of the organ (Mizukami and Fischer, 2000). This process is modulated by several factors and our data suggests that AN3 contributes to this process.

### AN3 Acts Maternally to Influence Seed Mass

The mass of a seed is modulated through the coordinated growth of maternal sporophytic and zygotic tissues. Reciprocal cross experiments between *an3-4* and Col-0 plants were performed to determine whether *AN3* functioned maternally or zygotically. When pollen of Col-0 and *an3-4* was used as the donor and Col-0 plants were used as the acceptor, the seed mass resulting from the reciprocal crosses was not altered with the change of the donor (Col-0 and *an3-4*) (**Supplementary Table S1**). Similarly, when pollen of Col-0 and *an3-4* was used as the donor and *an3-4* plants were used as the acceptor, the seed mass of reciprocal crosses were also not altered with the change of the donor (Col-0 and *an3-4*) (**Supplementary Table S1**). On the contrary, with different acceptors (Col-0 and *an3-4*), the seed mass of reciprocal crosses was altered. This phenomenon indicates that in *an3-4* mutant, the integument (the integument of *Arabidopsis* finally forms seed coat) determines the seed size (a maternal effect). Moreover, it is obvious that the genotype of the seed may be not relevant in this case of the maternal effect. These above findings suggest that *an3-4* seed mass is determined by maternal patterns.

We also observed the embryo sac or seed cavity at 4 DAP in *an3-4* and Col-0 plants. Cytological observations indicated that the average area of the ovule in *an3-4* plants was ∼1.38 times that of wild-type (**Supplementary Figures S3A–C**). In a similar fashion, the average area of embryos from *an3-4* plants was ∼1.40 times that of wild-type (**Supplementary Figures S3A,B,D**). These results indicate that the ovule and embryo in an *an3-4* mutant is grown coordinately. Thus, the changes in seed mass maternally controlled by AN3 were reflected in the size of the embryo. On average, the cell area of the *an3-4* maternal integument was ∼1.72 times that of wild-type (**Supplementary Figures S3A,B,E**). Similar with this, on average, the epidermal cell size of *an3-4* embryos was ∼1.70 times that of wild-type (**Figures 3B,D**). These data (i.e., 1.40/1.72 = 0.8) indicated that the increased embryo (or integument) size of *an3-4* plants may be due to embryo (or integument) cell elongation. Therefore, we concluded that the *an3-4* mutant had a maternal influence on the regulation of seed mass, which agrees with a recent report (Lee et al., 2014). Hence, with the ovules matured, *AN3/GIF1* expression is strongly detected in the chalazal portions of the ovule and in the initiating and developing integuments.

When cells in the outermost layer of the integument were counted, Lee et al. (2014) found that the *gif1/an3* mutants had significantly decreased cell numbers, whereas integument mass of *gif1/an3* but not *gif1gif2gif3* plants was increased. This suggested that the cell size of *gif1/an3* integuments was enhanced. These results further imply that AN3/GIF1 is involved in the regulation of integument development, which is consistent with the notion that the absence of AN3 activity has a maternal influence on the regulation of seed mass. Similar with AN3, KLU which encodes a cytochrome P450 and maternally influences seed mass, is expressed in the inner integument of developing ovules, where it non-cell autonomously stimulates cell proliferation, therefore determining the growth potential of both the seed coat and the seed (Adamski et al., 2009).

In a similar fashion to AN3, other reports (Garcia et al., 2005; Luo et al., 2005) have also showed the impact of maternal functions on seed mass. In the transparent *testa glabra2* (*ttg2*) mutant, for example, endosperm growth is restricted as a consequence of prevention of cell elongation in the integument and is one of the few examples of maternal control of seed size in *Arabidopsis* (Garcia et al., 2005). The size of cells of the outer integument was correlated with embryo size in the *mini3* mutant (Luo et al., 2005). Consequently, the *mini3* mutant showed smaller seed mass. As embryo cell size is not altered in the mutant but the overall size of the embryo is smaller, the seed must contain fewer cells (Luo et al., 2005).

### GRF1 Is Not Involved in Regulating *MINI3* Expression during Seed Development

*AN3/GIF1* and *GRF1* regulate the growth and shape of leaf blades and petals through modulating cell proliferation (Kim and Kende, 2004). Therefore, we investigated whether AN3/GIF1 might act as a cofactor and interact with the GRF1 protein that is a specific transcription factor for the *MINI3* promoter and mediate regulation of *MINI3* transcription during seed development. *grf1* plants had similar cotyledon size (**Supplementary Figures S1A,B**), seed mass (**Figures 1A–E**), seed/silique number, silique length, seed/silique weight, flower number, elongation silique number, total seed/plant weight (**Figures 2A–F**) and cotyledon embryo area (**Figures 3A,C**) to wild-type. In addition, expression levels in the developing siliques of *SHB1, MINI3, IKU2*, and *AP2* did not significantly differ between wild-type and *grf1* mutant plants (**Figure 5A**). These results suggest that, at least, AN3/GIF1 might act as a cofactor and interact with one or more unknown proteins that might function as specific transcription factors to regulate *MINI3* transcription during seed development. However, our results suggest that this interacting protein is not GRF1.

### AN3 Acts Genetically Upstream of MINI3 in Regulating Seed Mass

**FIGURE 7:**
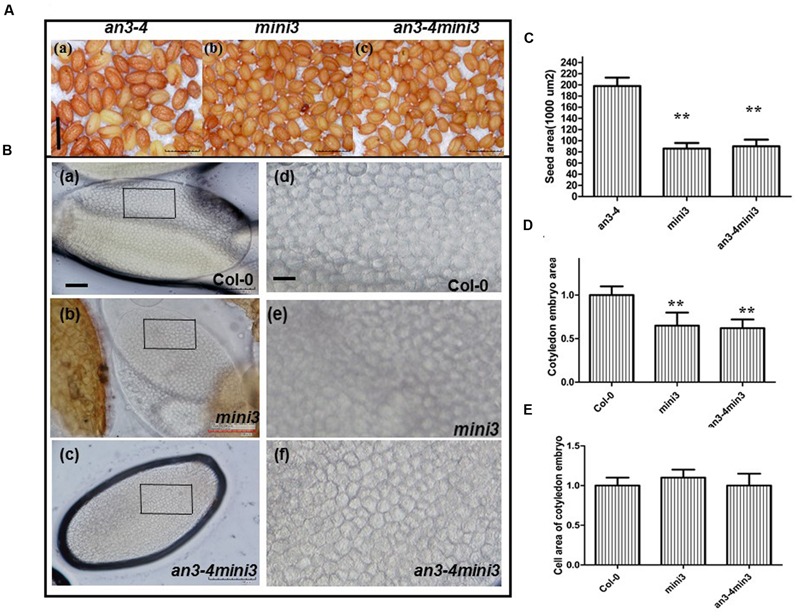
***AN3* acts genetically upstream of *MINI3* in regulating seed mass. (A)** Representative mature seeds of *an3-4, mini3*, and *an3-4 mini3* plants, respectively. Bar = 0.1 mm. **(B)** Representative mature embryos of Col-0 and *mini3* plants were isolated and visualized, respectively. Pane in (a–c) was amplified to (d–f). Bars = 100 μm in (a–c); Bars = 10 μm in (d–f). **(C)** Bar graph exhibiting differences in seed area between *an3-4, mini3*, and *an3-4 mini3* mutants. Data are means ± SD from at least five independently propagated lines (*n* = 22). **(D)** and **(E)** Bar graph exhibiting the difference in embryo area **(D)** and embryo cell area **(E)** between *mini3* and Col-0 seeds. Col-0 is set as 1.0. Data are means ± SD from at least five independently propagated Col-0 and mutant lines (^∗∗^*P* < 0.01; in **D**, *n* = 15; in **E**, *n* = 32).

We performed double-mutant analysis, crossing *an3-4*, which has large seed mass, with *mini3-2*, which has small seed mass, to determine whether *AN3* acted upstream of *MINI3* in controlling this trait. Thus, we selected the genotypes from the segregating *F*_3_ population identified by PCR genotyping. *mini3-2* obviously reduced the large seed phenotype of *an3-4* (**Figures 7A,C**). The *an3-4 mini3-2* double mutant had very similar seed mass to the *mini3-2* single mutant (**Figures 7A,C**), indicating that *mini3-2* was epistatic to *an3-4*. These genetic results confirmed that AN3 acted upstream of MINI3 in regulating seed mass.

### Reduced Seed Size Exhibited by *mini3* Plants Is Due to Reduced Embryo Cell Number

We isolated and visualized mature embryos originating from wild-type and *mini3* mutant seeds. The *mini3* mature seeds had less mass than wild-type (**Figures 7A,C**). Cytological experiments showed smaller average area of *mini3* cotyledon embryos than that of wild-type [**Figures 7B(a,b,d,e),D**]. However, the embryo cell size in *mini3* cotyledons did not differ from that of wild-type [**Figures 7B(a,b,d,e),E**]. Thus, the data indicates that, on average, the embryo cell size exhibited by *mini3* plants was not altered, rather embryo size reduction was due to the reduced number of embryo cells. Therefore, the absence of MINI3 resulted in a smaller embryo and fewer cells compared with wild-type. In addition, cytological experiments revealed a smaller average area of *an3-4 mini3* cotyledon embryos relative to wild-type [**Figures 7B(a,c,d,f),D**]. However, the embryo cell size in cotyledons from *an3-4mini3* plants was not significantly different from that of wild-type [**Figures 7B(a,c,d,f),E]**. Collectively, this data implies that the reduced seed size exhibited by *mini3* plants is due to reduced embryo cell number.

## Discussion

### AN3 Negatively Regulates *MINI3* Expression

AN3 is required for the expression of *MINI3* (**Figure 5**). Double-mutant analyses revealed that the effect of *an3* on seed mass was largely dependent on MINI3 function (**Figure 7**). *MINI3* encodes WRKY10, a WRKY class transcription factor. Levels of *MINI3* transcripts were higher in *an3-4* than in wild-type plants. We also found that *AN3* and *MINI3* had different expression patterns in many organs. While *AN3* was expressed in young leaves, flowers, stems, stamens, and embryos (**Figure 6**), expression of *MINI3* was not detected in these organs (Luo et al., 2005). *AN3* expression was also not detected in mature pollen grains (**Figure 6**) unlike *MINI3* (Luo et al., 2005).

The action of AN3 in regulating seed size is maternally mediated (**Supplementary Table S1**). This would suggest that AN3 acts by influencing integument growth and this idea is supported by the fact the ovules of *an3* plants are reported to be abnormally large, even before fertilization (Lee et al., 2014). However, the MINISEED3 protein has been described as acting zygotically to increase seed size through promotion of early endosperm expansion (Luo et al., 2005). Thus, AN3 may act maternally to repress early zygotic endosperm expansion driven by MINI3, which would explain the fact that the *mini3* phenotype is epistatic to the *an3* phenotype. In such a scenario epistasis would not necessitate a direct interaction of AN3 with the *MINI3* promoter. On the other hand, we find that AN3 acts to repress *MINI3* expression and indeed, binds the *MINI3* promoter. However, direct regulation of *MINI3* expression may not explain the maternal action of AN3. That is, the ChIP data indicated that AN3 is associated with *MINI3* promoter, AN3 acts in the endosperm or the embryo (**Figure 6**; Lee et al., 2014), where *MINI3* performs its function. Thus, the possible scenario to resolve the conflict is whether AN3 is a maternally imprinted gene expressed in the endosperm or the embryo. In fact, AN3 encodes a homolog of the human transcription coactivator SYT and is a putative transcription coactivator, synovial SYT (Horiguchi et al., 2005). In synovial sarcomas, chromosomal translocations of the SYT locus to the SSX locus are always observed (Horiguchi et al., 2005). Moreover, the chimeric SYT-SSX protein might possess changed regulatory functions, which can trigger tumor development (Horiguchi et al., 2005). A few reports suggested that the human transcription coactivator SYT might be an imprinted gene (Sun et al., 2006; Cironi et al., 2009). Thus, AN3 may be an imprinted gene. It will await future research to determine whether AN3 is a maternally imprinted gene expressed in the endosperm or the embryo of *Arabidopsis*.

Besides the above interpretation, we can provide other model, which is as followed in details. AN3 encodes a homolog of the human transcription coactivator SYT and is a putative transcription coactivator (Horiguchi et al., 2005). A similar result has been reported for many other proteins, such as FLOWERING LOCUS T (FT), GIGANTEA (GI), FLAVIN BINDING, KELCH REPEAT, F-BOX1 (FKF1), and SHB1 (Wigge et al., 2005; Sawa et al., 2007; Zhou et al., 2009). Although FT does not have DNA binding activity, it can associate with *AP1* promoters by a direct interaction with FLOWERING LOCUS D (a bZIP transcription factor) that directly binds to the *AP1* promoter (Wigge et al., 2005). In addition, GI and FKF1 can associate with CONSTANS promoters through a direct interaction with *CYCLING DOF FACTOR1* (a Dof transcription factor) in a blue light-dependent manner (Sawa et al., 2007). SHORT HYPOCOTYL UNDER BLUE1 (SHB1) might have a direct interaction with other unknown proteins that are specific transcription factors for *MINISEED3* (*MINI3*) and *HAIKU2* (*IKU2*) promoters and regulate transcription of *MINI3* and *IKU2* during the initial phase of seed development (Zhou et al., 2009).

**FIGURE 8:**
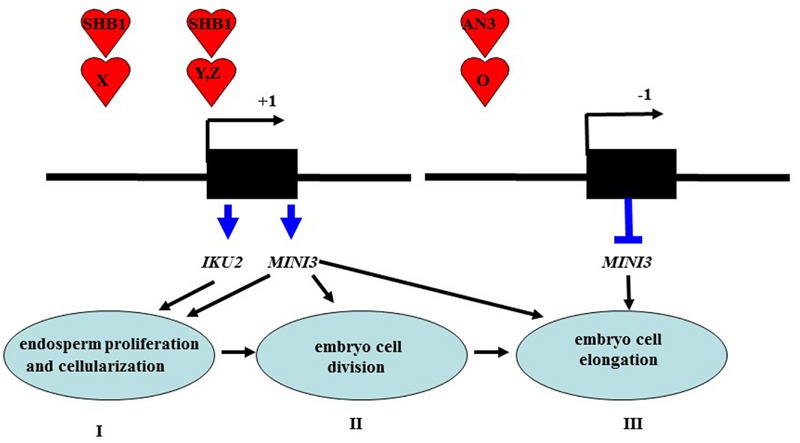
**A working model is proposed for AN3 function in seed development.** I stage: SHB1 is recruited to the promoters of *MINI3* and *IKU2* via protein X and modulates the expression of key genes including *MINI3* and *IKU2* that are required for endosperm proliferation and cellularization (Zhou et al., 2009). II stage: SHB1 deploys a similar mechanism to present itself by protein Y to the promoters of unknown targets including *Z* gene or *MINI3* that are required for embryo cell proliferation and elongation (Zhou et al., 2009). II stage: AN3 is recruited to the promoters of *MINI3* by protein O and negatively regulates the expression of key genes such as *MINI3* that are required for embryo cell division; which in turn triggers cell elongation (terms: complementation). *IKU2* is expressed uniquely in the endosperm (Luo et al., 2005), whereas *MINI3* is expressed in both the endosperm, the embryo and integument (Luo et al., 2005; Zhou et al., 2009) in this work, we found that AN3 is expressed in the embryo and integument but not in the endosperm.

While AN3 was also associated with the *MINI3* promoter *in vivo*, AN3 might not have a direct interaction with SHB1 because AN3 is a transcription coactivator not a transcription factor. Similarly, AN3 probably acts as a transcription coactivator and might interact with other unknown proteins that are specific transcription factors for the *MINI3* promoter to regulate the transcription of this gene during seed mass development (**Figure 8**). In this context, GRF1 encodes a putative transcription factor, which interacts with AN3 to promote cell proliferation (Kim and Kende, 2004; Horiguchi et al., 2005). Therefore, AN3 might have a direct interaction with GRF1 that is a specific transcription factor and subsequently the AN3–GRF1 complex might directly bind to the *MINI3* promoter regulating transcription of this target gene during modulation of seed mass. However, we found that *grf1* plants had similar cotyledon size (**Supplementary Figures S1A,B**), seed mass (**Figures 1A–E**), seed number, silique length, seed weight, flower number, elongation silique number, total seed weight (**Figures 2A–F**), and cotyledon embryo area (**Figures 3A,C**) with those of wild-type. These results imply that *GRF1* is not a candidate.

### Regulation of Seed Embryo Development by Cell Division and Cell Elongation Mediated via an *AN3-MINI3* Cascade

AN3 acted maternally to influence seed mass, suggesting AN3 regulates seed mass by controlling cell proliferation of the seed coat (integument). However, the embryo constitutes the major volume of a mature seed in *Arabidopsis* and the changes in seed mass were reflected in the size of the embryos. Thus, we focused our attention on the embryo. As estimated from both embryo size and embryo cell size of *an3 mini3* double mutants, the cell number in *an3 mini3* embryos is expected to be almost identical to parental *mini3* mutants. That is, in this work, we used *mini3* [for *mini3-2* (SALK_050364)], and the cell number in the *mini3-2* mature embryo is reduced to ∼60% of wild-type (Col-0) (**Figure 7D**). Consistent with this result, Zhou et al. (2009) also showed that seed size (or embryo size) in the *mini3-2* mutant is reduced to ∼60% of wild-type (Col-0), whereas embryo cell size in this mutant is not altered. Embryo size is determined via both embryo cell size and cell number. Thus, the cell number in *mini3-2* mature embryos is reduced to ∼60% of wild-type (Col-0). Moreover, our findings indicated that the cell number in *an3-4 mini3-2* mature embryos was also reduced to ∼60% of wild-type (Col-0) (**Figure 7D**). However, cytological observations indicated that the average area of the *an3-4* cotyledon embryos was ∼1.40 times that of wild-type (**Figures 3B,D**). On average, the epidermal cell size of *an3-4* embryos was ∼1.70 times that of wild-type (**Figures 3B,D**). These data (i.e., 1.40/1.70 = 0.8) indicated that the increased embryo size of *an3-4* plants was due to embryo cell elongation.

These results also indicated that the cell number in *an3-4* mature embryos is reduced to ∼80% of wild-type (Col-0). This contrasts with *mini3-2* and *an3-4 mini3-2* plants where embryo cell number was only ∼60% of wild-type (Col-0). Therefore, the absence of *MINI3* function suppresses cell division in *an3-4* plants and also inhibits increased cell size during embryo development in this line. Larger cell size in *an3-4* plants is thought to result from a phenomenon termed compensation where larger *an3-4* cells result from a decrease in cell number which triggers an increase in mature cell size (Ferjani et al., 2007; Fujikura et al., 2007;). Thus, mutation of *MINI3* leads to a decrease of *an3-4 mini3-2* seed mass. These genetic findings indicate that AN3 mediated suppression of *MINI3* expression appears to occur at an early stage of seed embryo development (cell division), but is also influenced at a later stage of seed embryo development (cell elongation).

Consistent with genetic data, in seed embryo development, the expression of *AN3* can be detected in later embryo development (Kanei et al., 2012), whereas the expression of *MINI3* cannot be observed at this stage (Luo et al., 2005). Consistent with our molecular data that suggests AN3 trans-represses *MINI3* by associating with the *MINI3* promoter (**Figure 5**). Cell division activity, directly relevant for cell number, mainly occurs during the early phase of seed development. Conversely, cell elongation, relevant for cell size, mainly occurs during the late phase of seed development (Weber et al., 1996, 1997a,b). Thus, an *AN3-MINI3* gene cascade might regulate seed embryo development by controlling both cell division and cell elongation.

Compensation, such as that leading to larger cells in *an3-4* embryos, is thought to be largely independent from endoreduplication, although the underpinning molecular mechanism is unknown (Ferjani et al., 2007; Fujikura et al., 2007). Here, we find that mutation of *MINI3* suppresses cell division in an *an3-4* mutant during early embryo development and in turn inhibits the increased cell size phenotype (cell elongation) during later embryo development. Thus, our findings reveal that the larger epidermal cells in *an3-4* plants are due to compensation, which might occur as a result of the dysregulation of *MINI3* expression.

## Accession Numbers

Sequence data derived from this paper can be found in the *Arabidopsis* Genome Initiative database under the following accession numbers *AN3* (At5G28640) and *MINI3* (At1G55600).

## Author Contributions

L-SM designed experiments. L-SM performed the experiments. L-SM, and Y-BW completed statistical analysis of data. L-SM, GL, and J-HJ wrote, edited and revised this manuscript.

## Conflict of Interest Statement

The authors declare that the research was conducted in the absence of any commercial or financial relationships that could be construed as a potential conflict of interest.

## Footnotes

1 http://www.olympus-global.com/

## Supplementary Material

FIGURE S1 ***an3-4* mutant plants had enlarged cotyledons. (A)** Representative 10-day-old mature cotyledons of an3-4, *35S-AN3/an3-4, grf1* and wild-type (Col-0) grown on solid MS medium with 1% sucrose, respectively. Bar = 5 mm. **(B)** Bar graph showing the difference in the cotyledon area between *an3-4, 35S-AN3/an3-4, grf1*, and wild-type (Col-0). Data are means ± SD from at least 10 independently propagated lines of *an3-4, 35S-AN3/an3-4, grf1* and Col-0 (^∗∗^*P* < 0.01; *n* = 20). Col-0 is set as 1.0.

FIGURE S2 ***an3-4* mutant plants present shorter stamens than wild-type (Col-0).** Arrows indicate stamen. Scale bars: 1 mm

FIGURE S3 **AN3 regulates embryo size by modulation of integument. (A)** Col-0 (Col-0 embryo) × Col-0 (maternal sporophytic tissue). **(B)** *an3-4* (*an3-4* embryo) × *an3-4* (maternal sporophytic tissue). Scale bars: **(A)**, **(B)**, 100 μm. **(C)** Bar graph exhibiting the difference in ovule area between *an3-4* and Col-0 seeds. Data are means ± SD from at least five independently propagated lines (*n* = 12). **(D)** and **(E)** Bar graph exhibiting the difference in global embryo area **(D)** and integument cell area **(E)** between *an3* and Col-0 seeds. Col-0 is set as 1.0. Data are means ± SD from at least five independently propagated Col-0 and mutant lines (^∗∗^*P* < 0.01; in **D**, *n* = 10; in **E**, *n* = 38).

TABLE S1 ***an3-4* act maternally for regulating seed mass**.

TABLE S2 **primers in this work are used**.
