# Corrigendum: Seed Embryo Development Is Regulated via an *AN3-MINI3* Gene Cascade

**DOI:** 10.3389/fpls.2017.01073

**Published:** 2017-06-14

**Authors:** Lai-Sheng Meng, Yi-Bo Wang, Gary J. Loake, Ji-Hong Jiang

**Affiliations:** ^1^The Key Laboratory of Biotechnology for Medicinal Plant of Jiangsu Province, School of Life Science, Jiangsu Normal UniversityXuzhou, China; ^2^Transformational Centre for Biotechnology of Medicinal and Food Plants, Jiangsu Normal University – Edinburgh UniversityXuzhou, China; ^3^School of Bioengineering and Biotechnology, Tianshui Normal UniversityTianshui, China; ^4^Institute of Molecular Plant Sciences, School of Biological Sciences, Edinburgh UniversityEdinburgh, United Kingdom

**Keywords:** *ANGUSTIFOLIA3* (*AN3*), MINISEED*3* (*MINI3*), seed mass, embryo cell elongation, embryo cell division, *Arabidopsis*

In the original article, there was a mistake in the legend for [Figure 6] as published. **[We state the mistake which was made in A, B, H and J. Moreover, we added Bars]**. The correct legend appears below. The authors apologize for this error and state that this does not change the scientific conclusions of the article in any way.

**Figure 6:**
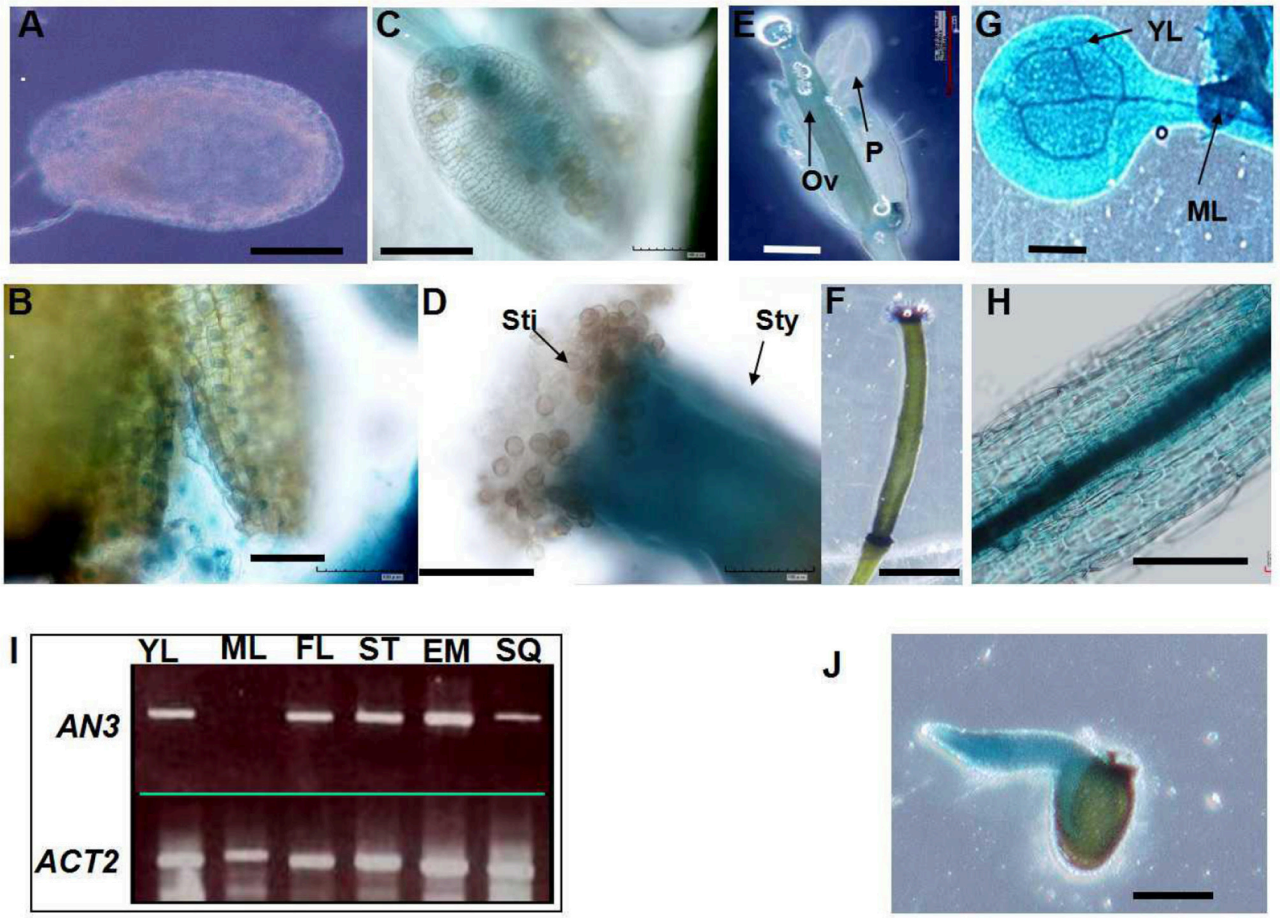
*AN3* expression analysis. Expression of *AN3pro:GUS* in the integument **(A)**, seed coat **(B)**, hypocotyl **(H)**, and embryos **(J)**. Bars = 50 μm for **(A–H)** and **(J)**.

In the original article, there was a mistake in [Figure 6] as published. **[We state the mistake which was made in G and F]**. The corrected [Figure 6] appears below. The authors apologize for this error and state that this does not change the scientific conclusions of the article in any way.

In the original article [Kawade, K., Horiguchi, G., and Tsukaya, H. (2010). Non cellautonomously coordinated organ size regulation in leaf development. Development 137: 4221–4227] was not cited in the article. The citation has now been inserted in [RESULTS], [*an3-4* Plants Have Increased Seed Mass], [Paragraph Number 1] and should read:

***an3-4* plants have increased seed mass**

While investigating drought tolerance exhibited by *an3-4* plants, we observed this line had larger cotyledons than wild-type (Col-0). To confirm whether these large cotyledons were due to a lack of AN3 protein activity, we investigated the phenotype of *an3-4* complemented lines (Kawade et al., 2010).

The authors apologize for this error and state that this does not change the scientific conclusions of the article in any way.

In the original article, there was an error. [The mistake that was made in *AN3* Expression Pattern in RESULTS].

A correction has been made to [RESULTS], [AN3 Expression Pattern], [Paragraph 8]:

To further explore AN3 function, we examined the expression pattern of the β-glucuronidase (GUS) reporter gene driven by a native AN3 promoter. Analysis of *Arabidopsis* transformed with an approximately 2.0-kb *AN3* promoter–GUS reporter gene fusion (*AN3pro:GUS*) showed that *AN3pro:GUS* was expressed in integument and seed coat (Figures 6A,B), stigmas (Figure 6D), siliques, young leaves and hypocotyls (Figures 6F–H) and embryos (Figure 6J), but not in mature pollen grains (Figure 6C) petals (Figure 6E), mature leaves (Figure 6G). Consistent with this, RT-PCR results indicated the presence of *AN3* transcripts in the corresponding organs (Figure 6I).

In the original article, there was an error. [The mistake that was made in *an3-4* Plants Have Increased Seed Mass in RESULTS].

A correction has been made to [RESULTS], [*an3-4* Plants Have Increased Seed Mass], [Paragraph 1]:

These lines harboring a *35S:AN3* transgene in an *an3-4* genetic background exhibited similar cotyledon size to Col-0 (Supplementary Figures S1A,B). A *GRF1* loss-of-function mutant showed similar cotyledon size relative to wild-type (Supplementary Figures S1A,B), implying GRF1 may not be involved in the regulation of seed mass. Taken together our data suggests that the *an3-4* plants have larger cotyledons relative to wild-type (Col-0).

In the original article, there was an error. [The mistake that was made in The Large Seeds of *an3-4* Plants Are Due to Increased Embryo Cell Size in RESULTS].

A correction has been made to [RESULTS], [The Large Seeds of *an3-4* Plants Are Due to Increased Embryo Cell Size], [Paragraph 4]:

However, these parameters were similar in *grf1* plants relative to wild-type (Figures 3A,C). Cytological observations indicated that the average area of cotyledon embryos from *an3-4* plants was about 1.40 times that of wild-type (Figures 3B,D).

The authors apologize for this error and state that this does not change the scientific conclusions of the article in any way.

## Conflict of interest statement

The authors declare that the research was conducted in the absence of any commercial or financial relationships that could be construed as a potential conflict of interest.

