# Supplementary material for: Seed Embryo Development Is Regulated via an AN3-MINI3 Gene Cascade
Source: Front Plant Sci. 2016 Nov 3;7:1645. doi: 10.3389/fpls.2016.01645 (PMC5093144; doi:10.3389/fpls.2016.01645)
Supplement: FIGURE S1 — an3-4 mutant plants had enlarged cotyledons. (A) Representative 10-day-old mature cotyledons of an3-4, 35S-AN3/an3-4, grf1 and wild-type (Col-0) grown on solid MS medium with 1% sucrose, respectively. Bar = 5 mm. (B) Bar graph showing the difference in the cotyledon area between an3-4, 35S-AN3/an3-4, grf1, and wild-type (Col-0). Data are means ± SD from at least 10 independently propagated lines of an3-4, 35S-AN3/an3-4, grf1 and Col-0 (∗∗P < 0.01; n = 20). Col-0 is set as 1.0. [file Data_Sheet_1.pdf]

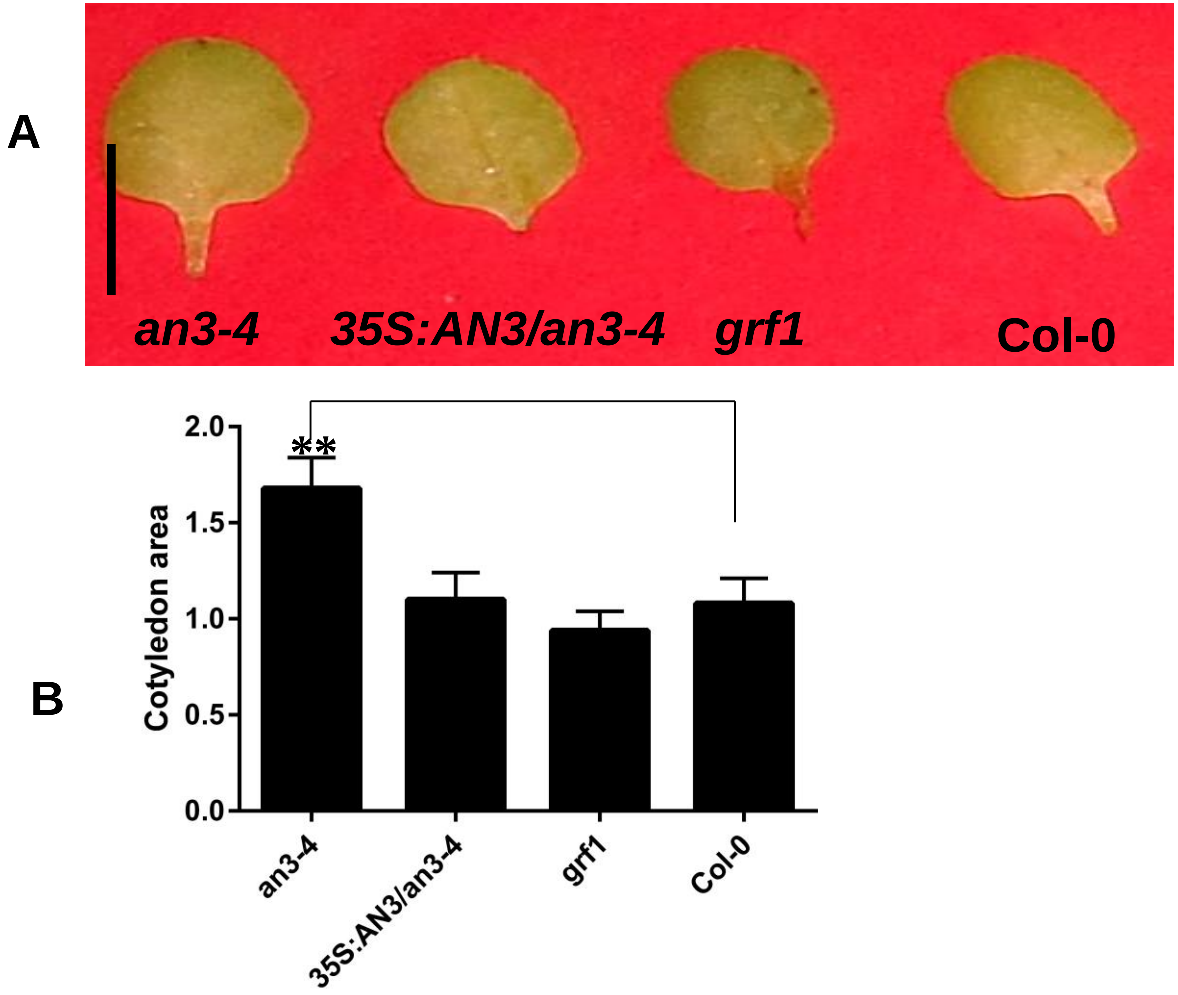

Supplemental Figure 1. *an3-4* Mutant Plants Had Enlarged Cotyledons.

(A) Representative 10-d-old mature cotyledons of *an3-4*, *35S-AN3/an3-4*, *grf1* and wild-type (Col-0) grown on solid MS medium with 1% sucrose, respectively. Bar = 5 mm.

(B) Bar graph showing the difference in the cotyledon area between *an3-4*, *35S-AN3/an3-4*, *grf1* and wild-type (Col-0). Data are means ± SD from at least 10 independently propagated lines of *an3-4*, *35S-AN3/an3-4*, *grf1* and Col-0 (** indicates $P < 0.01$; n=20). Col-0 is set as 1.0.

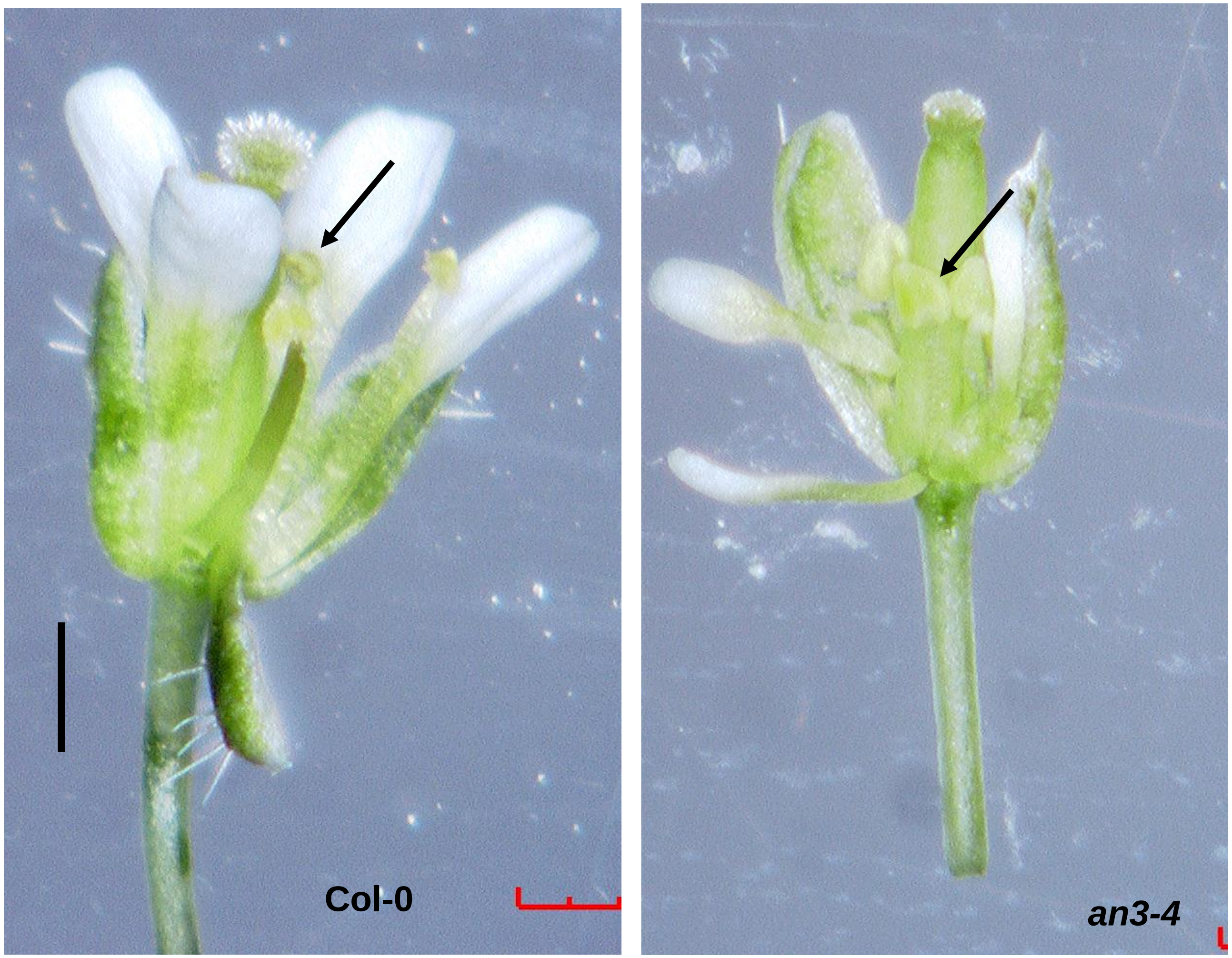

Supplemental Figure 2. *an3-4* plants present shorter stamens than do wild-type (Col-0). Arrows indicate stamen. Scale bars: 1 mm

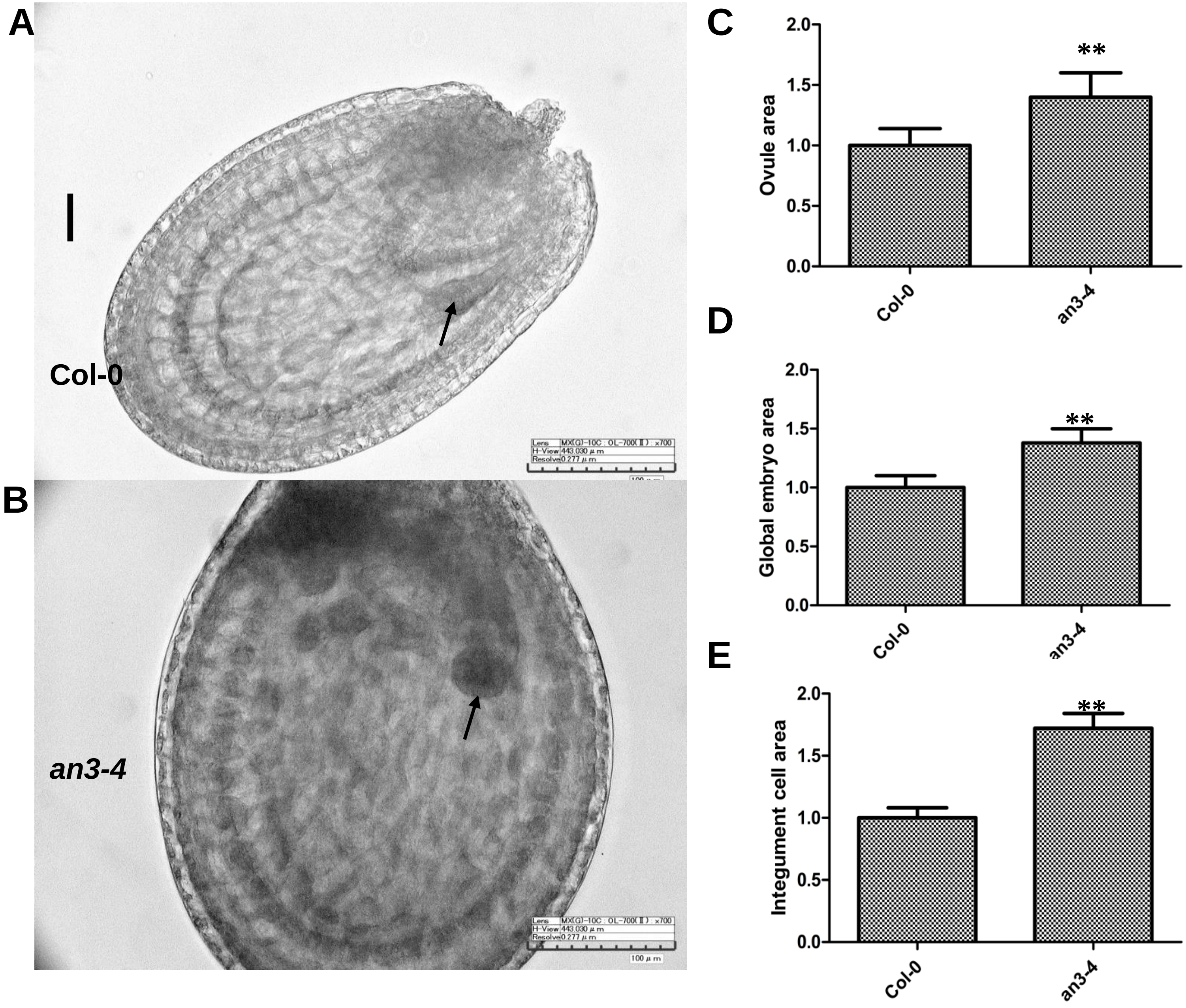
A
Col-0
B
an3-4
C
Ovule area
Col-0
an3-4
**
D
Global embryo area
Col-0
an3-4
**
E
Integument cell area
Col-0
an3-4
**
100 µm

Supplemental Figure 3. AN3 regulates embryo size by modulation of integument.
A. Col-0 (Col-0 embryo) X Col-0 (maternal sporophytic tissue).
B. an3-4 (an3-4 embryo) X an3-4 (maternal sporophytic tissue).
Scale bars: A, B, 100 um
C. Bar graph exhibiting the difference in ovule area between an3-4 and Col-0 seeds. Data are means ± SD from at least five independently propagated lines (n= 12).
D and E. Bar graph exhibiting the difference in global embryo area (D) and integument cell area (E) between an3 and Col-0 seeds. Col-0 is set as 1.0. Data are means ± SD from at least five independently propagated Col-0 and mutant lines (** indicates $P < 0.01$; in D, n=10; in E, n=38).

Table s1: *an3-4* act maternally for regulating seed mass.

parent		Seed weight
female	male	
Wild type (Col-0)	Wild type (Col-0)	1.8 ± 0.1 mg (100seeds)
Wild type (Col-0)	*an3-4*	1.8 ± 0.2
an3-4	Wild type (Col-0)	2.9 ± 0.1
an3-4	*an3-4*	2.9 ± 0.2

Reciprocal crosses between wild-type plants and *an3-4* mutants were performed on secondary inflorescences. Plants were grown together in the same conditions, and were manually pollinated. Means ± SD are shown. For each data point, at least 5 seedlings were measured.

Table S2

(for Q-PCR) AP2 F-5'-ATGTGGGATCTAAACGACGC-3' and R-5'-ACAAAACTTAACACCAAACCAGT-3';
(for Q-PCR) SHB1 F-5'-CATCCAAGCTTCCCGGAATAGGTCA-3' and R-5'-CCGCCGTCTCGAGCCCTTCT-3'
(for Q-PCR) IKU2 F-5'-GGTGTCCGGAGAGTTCCCACGA-3' and R-5'-CGCTCATGCAGCTGCTCCCA-3'
(for Q-PCR) AN3: F-5'-GCCTCAGCCACCAAGTGTGCAT-3' and R-5'-ACCGCCACCACCACTTCCCA-3'
(for Q-PCR) AN3 F-5'-AACGCAAAACAACTTTTGTGGA-3' and R-5'-TCAGAG AAATAACATATTAAGATTA -3'
(for AN3-GUS) Pro-AN3-(P1- ggggacaagtttgtacaaaaaagcaggctTTTGTAAGCGTTTCAGAATCCT and P2- ggggaccactttgtacaagaaagctgggtTAACTATTGAAGATGTGTATCTC)
(for pMD111-MINI3:GFP) Pro-MINI3-(P1- ggggacaagtttgtacaaaaaagcaggct aaacggagcatcatcgtccaa and P2- ggggaccactttgtacaagaaagctgggt tcttatatagcaaagtttgtaacc)
(for pCB2004-35S-AN3) ggg gac aag ttt gta caa aaa agc agg ct ATG CAA CAG CAC CTG ATG CAG AT; ggg gac cac tttg tac aag aaa gct ggg t TCA ATT CCC ATC ATC TGA TGA TTT C
(for ChIP) M1 (F1-ttttacttaaaccgaaccgaagta and R1- tcttatatagcaaagtttgtaacc)
(for ChIP) 2 (F2- aataaatatatgatcacggtgatat and R2- attccaattaattttagaccatatt)
(for ChIP) M3 (F3- aaacggagcatcatcgtccaa and R3- aaccaacttttaaataaactcgat)
(for ChIP) CDS (F-ATGAGTGATTTTGATGAAAACTTCA and R-GAATCCTGGAGAGATTGCAACG)
